# (*S*)-2-[(2,4-Dichloro­phen­yl)(hy­droxy)meth­yl]-5,5-dimethyl-1,3,2-dioxa­phosphinane 2-oxide

**DOI:** 10.1107/S1600536811009585

**Published:** 2011-03-19

**Authors:** Chubei Wang, Hao Peng, Xiaosong Tan, Hongwu He

**Affiliations:** a Key Laboratory of Pesticide and Chemical Biology, College of Chemistry, Central China Normal University, Wuhan 430079, People’s Republic of China

## Abstract

In the title mol­ecule, C_12_H_15_Cl_2_O_4_P, the cyclic dioxaphosphinane ring adopts a chair conformation. In the crystal, inter­molecular O—H⋯O hydrogen bonds link the mol­ecules into chains propagating along the *b* axis.

## Related literature

For the synthesis and biological activity of hy­droxy­dioxa­phos­phinane derivatives, see: Peng *et al.* (2007 ▶); Liu *et al.* (2006 ▶). For the synthesis of chiral cyclic hy­droxy­dioxa­phos­phin­anes, see: Zhou *et al.* (2008 ▶).
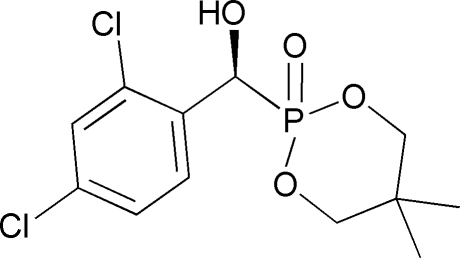


## Experimental

### 

#### Crystal data

C_12_H_15_Cl_2_O_4_P
*M* *_r_* = 325.11
Monoclinic, 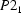

*a* = 7.0263 (9) Å
*b* = 9.9443 (13) Å
*c* = 10.6462 (14) Å
β = 93.975 (2)°
*V* = 742.08 (17) Å^3^
*Z* = 2
Mo *K*α radiation
μ = 0.55 mm^−1^
*T* = 298 K
0.16 × 0.12 × 0.10 mm

#### Data collection

Bruker SMART APEX CCD area-detector diffractometer
4069 measured reflections
2597 independent reflections
2478 reflections with *I* > 2σ(*I*)
*R*_int_ = 0.067

#### Refinement

*R*[*F* ^2^ > 2σ(*F* ^2^)] = 0.042
*wR*(*F* ^2^) = 0.105
*S* = 1.01
2597 reflections
177 parameters
1 restraint
H atoms treated by a mixture of independent and constrained refinement
Δρ_max_ = 0.39 e Å^−3^
Δρ_min_ = −0.25 e Å^−3^
Absolute structure: Flack (1983 ▶), 1140 Friedel pairs
Flack parameter: −0.15 (8)

### 

Data collection: *SMART* (Bruker, 2001 ▶); cell refinement: *SAINT-Plus* (Bruker, 2001 ▶); data reduction: *SAINT-Plus*; program(s) used to solve structure: *SHELXS97* (Sheldrick, 2008 ▶); program(s) used to refine structure: *SHELXL97* (Sheldrick, 2008 ▶); molecular graphics: *PLATON* (Spek, 2009 ▶); software used to prepare material for publication: *PLATON*.

## Supplementary Material

Crystal structure: contains datablocks global, I. DOI: 10.1107/S1600536811009585/cv5059sup1.cif

Structure factors: contains datablocks I. DOI: 10.1107/S1600536811009585/cv5059Isup2.hkl

Additional supplementary materials:  crystallographic information; 3D view; checkCIF report

## Figures and Tables

**Table 1:** Hydrogen-bond geometry (Å, °)

*D*—H⋯*A*	*D*—H	H⋯*A*	*D*⋯*A*	*D*—H⋯*A*
O1—H1⋯O4^i^	0.80 (5)	1.89 (5)	2.686 (3)	173 (4)

Symmetry code: (i) 
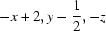
.

**Fig. 1.**
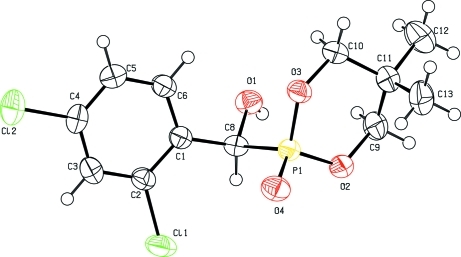
Molecular structure of (I), with displacement ellipsoids drawn at the 50% probability level. Hydrogen atoms are shown as spheres of arbitrary radius.

**Fig. 2.**
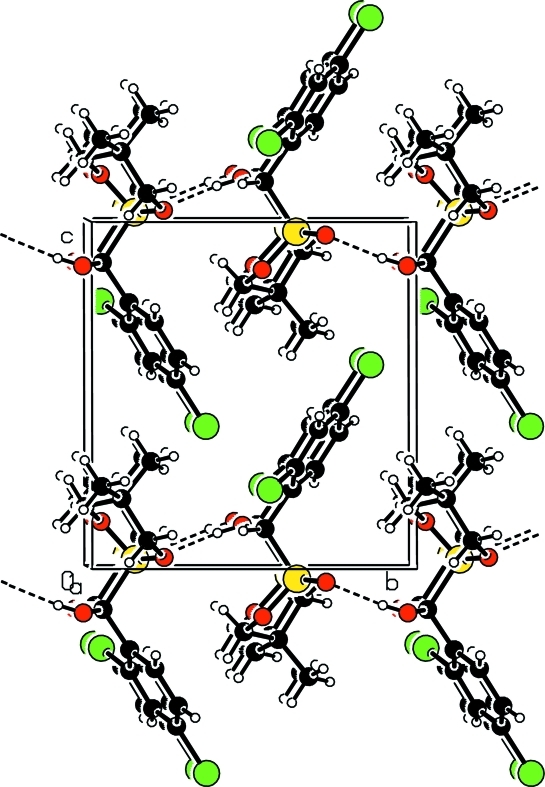
Part of the crystal packing, showing the intermolecular O—H···O hydrogen bonds as dashed lines.

## supplementary crystallographic information

### Comment

The cyclic alpha-hydroxydioxaphosphinanes exhibit various biological activities (Peng *et al.*, 2007; Liu *et al.*, 2006). The title compound, (I), is a chiral cyclic hydroxydioxaphosphinane derivative. Herewith we present its crystal structure.

In (I)  (Fig. 1), the cyclic dioxaphosphinane ring adopts a chair conformation. In the crystal structure, intermolecular O—H···O hydrogen bonds (Table 1) link the molecules into chains propagated along *b* axis (Fig. 2).

### Experimental

The title compound was prepared according to the known procedure (Zhou *et al.*, 2008). Diethylaluminum chloride (1 mmol) was added to a solution of (*S*,*E*)-2-(adamantan-1-yl)-4- (*tert*-butyl)-6(((1-hydroxy-3-methylbutan-2-yl)imino)methyl)phenol (1 mmol) in dichloromethane, The mixture was stirred at room temperature for 1 h. The aldehyde and cyclic phosphite was added and the mixture was stirred for another 2 h. The reaction was quenched by diluted hydrochloride acid. The pure title compound was afforded by column chromatography on silica gel (acetone/petroleum ether 1:2). Recrystallization from ethyl acetate over a period of one week gave colourless crystals of the title compound.

### Refinement

C-bound H atoms were geometrically positioned (C—H 0.93–0.98 Å) and refined as riding, with *U*_iso_(H) = 1.2-1.5 *U*_eq_(C). O-bound H atom was located on a difference map and refined as riding (*U*_iso_(H) = 1.5*U*_eq_(O)) with O—H bond length restrained to 0.80 (4) Å.

### Crystal data

**Table:**

C_12_H_15_Cl_2_O_4_P	*F*(000) = 336
*M**_r_* = 325.11	*D*_x_ = 1.455 Mg m^−^^3^
Monoclinic, *P*2_1_	Mo *K*α radiation, λ = 0.71073 Å
*a* = 7.0263 (9) Å	Cell parameters from 2185 reflections
*b* = 9.9443 (13) Å	θ = 2.8–28.1°
*c* = 10.6462 (14) Å	µ = 0.55 mm^−^^1^
β = 93.975 (2)°	*T* = 298 K
*V* = 742.08 (17) Å^3^	Block, colourless
*Z* = 2	0.16 × 0.12 × 0.10 mm

### Data collection

**Table:**

Bruker SMART APEX CCD area-detector diffractometer	2478 reflections with *I* > 2σ(*I*)
Radiation source: fine-focus sealed tube	*R*_int_ = 0.067
graphite	θ_max_ = 25.5°, θ_min_ = 1.9°
φ and ω scans	*h* = −8→8
4069 measured reflections	*k* = −11→12
2597 independent reflections	*l* = −12→11

### Refinement

**Table:**

Refinement on *F*^2^	Secondary atom site location: difference Fourier map
Least-squares matrix: full	Hydrogen site location: inferred from neighbouring sites
*R*[*F*^2^ > 2σ(*F*^2^)] = 0.042	H atoms treated by a mixture of independent and constrained refinement
*wR*(*F*^2^) = 0.105	*w* = 1/[σ^2^(*F*_o_^2^) + (0.0604*P*)^2^] where *P* = (*F*_o_^2^ + 2*F*_c_^2^)/3
*S* = 1.01	(Δ/σ)_max_ < 0.001
2597 reflections	Δρ_max_ = 0.39 e Å^−^^3^
177 parameters	Δρ_min_ = −0.25 e Å^−^^3^
1 restraint	Absolute structure: Flack (1983), 1140 Friedel pairs
Primary atom site location: structure-invariant direct methods	Flack parameter: −0.15 (8)

### Special details

**Table:**

Geometry. All e.s.d.'s (except the e.s.d. in the dihedral angle between two l.s. planes) are estimated using the full covariance matrix. The cell e.s.d.'s are taken into account individually in the estimation of e.s.d.'s in distances, angles and torsion angles; correlations between e.s.d.'s in cell parameters are only used when they are defined by crystal symmetry. An approximate (isotropic) treatment of cell e.s.d.'s is used for estimating e.s.d.'s involving l.s. planes.
Refinement. Refinement of *F*^2^ against ALL reflections. The weighted *R*-factor *wR* and goodness of fit *S* are based on *F*^2^, conventional *R*-factors *R* are based on *F*, with *F* set to zero for negative *F*^2^. The threshold expression of *F*^2^ > σ(*F*^2^) is used only for calculating *R*-factors(gt) *etc*. and is not relevant to the choice of reflections for refinement. *R*-factors based on *F*^2^ are statistically about twice as large as those based on *F*, and *R*- factors based on ALL data will be even larger.

### Fractional atomic coordinates and isotropic or equivalent isotropic displacement parameters (Å^2^)

**Table:**

	*x*	*y*	*z*	*U*_iso_*/*U*_eq_	
C1	1.0374 (4)	0.6151 (3)	0.2326 (3)	0.0326 (6)	
C2	1.2166 (4)	0.6199 (3)	0.2946 (3)	0.0353 (6)	
C3	1.2552 (5)	0.6911 (4)	0.4050 (3)	0.0426 (7)	
H3	1.3771	0.6916	0.4451	0.051*	
C4	1.1093 (5)	0.7608 (3)	0.4537 (3)	0.0432 (8)	
C5	0.9289 (5)	0.7620 (4)	0.3937 (3)	0.0454 (8)	
H5	0.8316	0.8117	0.4264	0.054*	
C6	0.8951 (4)	0.6891 (3)	0.2856 (3)	0.0395 (7)	
H6	0.7728	0.6890	0.2461	0.047*	
C8	0.9910 (4)	0.5319 (3)	0.1163 (3)	0.0330 (6)	
H8	1.0894	0.4627	0.1112	0.040*	
C9	0.7747 (5)	0.4722 (4)	−0.1830 (3)	0.0461 (8)	
H9A	0.7300	0.4104	−0.1209	0.055*	
H9B	0.7880	0.4222	−0.2601	0.055*	
C10	0.6092 (4)	0.6611 (4)	−0.0866 (3)	0.0439 (8)	
H10A	0.5200	0.7344	−0.1037	0.053*	
H10B	0.5575	0.6030	−0.0242	0.053*	
C11	0.6301 (5)	0.5816 (4)	−0.2073 (3)	0.0456 (8)	
C12	0.4368 (6)	0.5156 (6)	−0.2449 (5)	0.0766 (14)	
H12A	0.4470	0.4632	−0.3199	0.115*	
H12B	0.3418	0.5841	−0.2605	0.115*	
H12C	0.4008	0.4583	−0.1780	0.115*	
C13	0.6874 (6)	0.6734 (5)	−0.3136 (3)	0.0617 (11)	
H13A	0.8071	0.7157	−0.2893	0.093*	
H13B	0.5913	0.7411	−0.3297	0.093*	
H13C	0.6996	0.6211	−0.3884	0.093*	
Cl1	1.40915 (11)	0.53739 (10)	0.23277 (9)	0.0563 (3)	
Cl2	1.15586 (17)	0.85446 (12)	0.59048 (10)	0.0703 (3)	
O1	0.8112 (3)	0.4671 (2)	0.1240 (2)	0.0404 (5)	
H1	0.818 (6)	0.390 (5)	0.102 (4)	0.061*	
O2	0.9609 (3)	0.5257 (2)	−0.1376 (2)	0.0425 (5)	
O3	0.7922 (3)	0.7156 (2)	−0.0361 (2)	0.0390 (5)	
O4	1.1503 (3)	0.7162 (2)	−0.0359 (2)	0.0450 (6)	
P1	0.98124 (10)	0.63058 (8)	−0.02830 (7)	0.0317 (2)	

### Atomic displacement parameters (Å^2^)

**Table:**

	*U*^11^	*U*^22^	*U*^33^	*U*^12^	*U*^13^	*U*^23^
C1	0.0314 (14)	0.0335 (17)	0.0328 (14)	−0.0005 (13)	0.0022 (11)	0.0054 (13)
C2	0.0315 (14)	0.0363 (16)	0.0380 (15)	0.0030 (14)	0.0008 (11)	0.0036 (14)
C3	0.0403 (18)	0.0474 (18)	0.0386 (17)	−0.0026 (15)	−0.0067 (13)	0.0031 (15)
C4	0.055 (2)	0.0433 (19)	0.0312 (16)	−0.0031 (16)	0.0021 (14)	−0.0047 (14)
C5	0.0400 (19)	0.051 (2)	0.0456 (19)	0.0057 (16)	0.0073 (15)	−0.0045 (16)
C6	0.0305 (16)	0.0443 (18)	0.0433 (18)	0.0024 (14)	−0.0014 (13)	−0.0034 (15)
C8	0.0277 (13)	0.0318 (15)	0.0397 (16)	0.0010 (13)	0.0031 (12)	0.0010 (13)
C9	0.0453 (18)	0.0470 (19)	0.0452 (19)	−0.0084 (16)	−0.0034 (14)	−0.0094 (16)
C10	0.0304 (15)	0.055 (2)	0.0464 (18)	0.0036 (14)	0.0009 (13)	−0.0034 (16)
C11	0.0402 (18)	0.056 (2)	0.0404 (17)	−0.0018 (16)	−0.0029 (13)	−0.0051 (16)
C12	0.048 (2)	0.099 (4)	0.080 (3)	−0.014 (3)	−0.0139 (19)	−0.019 (3)
C13	0.071 (3)	0.073 (3)	0.040 (2)	−0.001 (2)	−0.0067 (17)	0.0043 (18)
Cl1	0.0313 (4)	0.0680 (6)	0.0690 (6)	0.0119 (4)	−0.0002 (4)	−0.0118 (5)
Cl2	0.0797 (7)	0.0820 (7)	0.0480 (5)	−0.0019 (6)	−0.0050 (5)	−0.0268 (5)
O1	0.0354 (12)	0.0373 (12)	0.0489 (13)	−0.0063 (10)	0.0063 (9)	−0.0050 (11)
O2	0.0359 (11)	0.0502 (14)	0.0411 (12)	0.0067 (11)	0.0015 (9)	−0.0107 (11)
O3	0.0330 (12)	0.0408 (13)	0.0425 (12)	0.0059 (9)	−0.0020 (9)	−0.0058 (10)
O4	0.0364 (12)	0.0445 (13)	0.0544 (14)	−0.0067 (10)	0.0055 (10)	0.0060 (11)
P1	0.0292 (4)	0.0331 (4)	0.0327 (4)	0.0000 (3)	0.0020 (3)	−0.0015 (3)

### Geometric parameters (Å, °)

**Table:**

C1—C2	1.381 (4)	C9—H9B	0.9700
C1—C6	1.393 (4)	C10—O3	1.463 (4)
C1—C8	1.506 (4)	C10—C11	1.524 (5)
C2—C3	1.382 (5)	C10—H10A	0.9700
C2—Cl1	1.750 (3)	C10—H10B	0.9700
C3—C4	1.369 (5)	C11—C13	1.530 (5)
C3—H3	0.9300	C11—C12	1.536 (5)
C4—C5	1.379 (5)	C12—H12A	0.9600
C4—Cl2	1.741 (3)	C12—H12B	0.9600
C5—C6	1.366 (5)	C12—H12C	0.9600
C5—H5	0.9300	C13—H13A	0.9600
C6—H6	0.9300	C13—H13B	0.9600
C8—O1	1.425 (3)	C13—H13C	0.9600
C8—P1	1.822 (3)	O1—H1	0.80 (5)
C8—H8	0.9800	O2—P1	1.561 (2)
C9—O2	1.463 (4)	O3—P1	1.572 (2)
C9—C11	1.499 (5)	O4—P1	1.468 (2)
C9—H9A	0.9700		
			
C2—C1—C6	116.4 (3)	C11—C10—H10A	109.3
C2—C1—C8	123.4 (3)	O3—C10—H10B	109.3
C6—C1—C8	120.2 (2)	C11—C10—H10B	109.3
C1—C2—C3	122.9 (3)	H10A—C10—H10B	108.0
C1—C2—Cl1	120.4 (2)	C9—C11—C10	109.6 (3)
C3—C2—Cl1	116.7 (2)	C9—C11—C13	110.6 (3)
C4—C3—C2	118.2 (3)	C10—C11—C13	111.1 (3)
C4—C3—H3	120.9	C9—C11—C12	108.1 (3)
C2—C3—H3	120.9	C10—C11—C12	107.8 (3)
C3—C4—C5	121.2 (3)	C13—C11—C12	109.6 (3)
C3—C4—Cl2	119.0 (3)	C11—C12—H12A	109.5
C5—C4—Cl2	119.8 (3)	C11—C12—H12B	109.5
C6—C5—C4	119.1 (3)	H12A—C12—H12B	109.5
C6—C5—H5	120.5	C11—C12—H12C	109.5
C4—C5—H5	120.5	H12A—C12—H12C	109.5
C5—C6—C1	122.3 (3)	H12B—C12—H12C	109.5
C5—C6—H6	118.9	C11—C13—H13A	109.5
C1—C6—H6	118.9	C11—C13—H13B	109.5
O1—C8—C1	110.1 (2)	H13A—C13—H13B	109.5
O1—C8—P1	108.08 (19)	C11—C13—H13C	109.5
C1—C8—P1	113.1 (2)	H13A—C13—H13C	109.5
O1—C8—H8	108.5	H13B—C13—H13C	109.5
C1—C8—H8	108.5	C8—O1—H1	110 (3)
P1—C8—H8	108.5	C9—O2—P1	121.52 (19)
O2—C9—C11	111.9 (3)	C10—O3—P1	122.6 (2)
O2—C9—H9A	109.2	O4—P1—O2	112.27 (14)
C11—C9—H9A	109.2	O4—P1—O3	111.68 (14)
O2—C9—H9B	109.2	O2—P1—O3	106.63 (12)
C11—C9—H9B	109.2	O4—P1—C8	112.04 (13)
H9A—C9—H9B	107.9	O2—P1—C8	105.43 (14)
O3—C10—C11	111.6 (2)	O3—P1—C8	108.43 (13)
O3—C10—H10A	109.3		
			
C6—C1—C2—C3	1.5 (5)	O2—C9—C11—C12	−175.9 (3)
C8—C1—C2—C3	−176.6 (3)	O3—C10—C11—C9	56.3 (4)
C6—C1—C2—Cl1	−177.0 (2)	O3—C10—C11—C13	−66.1 (4)
C8—C1—C2—Cl1	5.0 (4)	O3—C10—C11—C12	173.8 (3)
C1—C2—C3—C4	−0.7 (5)	C11—C9—O2—P1	48.4 (4)
Cl1—C2—C3—C4	177.8 (3)	C11—C10—O3—P1	−44.1 (4)
C2—C3—C4—C5	−1.0 (5)	C9—O2—P1—O4	−153.1 (3)
C2—C3—C4—Cl2	−178.4 (2)	C9—O2—P1—O3	−30.4 (3)
C3—C4—C5—C6	1.8 (5)	C9—O2—P1—C8	84.7 (3)
Cl2—C4—C5—C6	179.3 (3)	C10—O3—P1—O4	151.7 (2)
C4—C5—C6—C1	−1.0 (5)	C10—O3—P1—O2	28.8 (3)
C2—C1—C6—C5	−0.6 (5)	C10—O3—P1—C8	−84.3 (3)
C8—C1—C6—C5	177.5 (3)	O1—C8—P1—O4	171.92 (19)
C2—C1—C8—O1	137.8 (3)	C1—C8—P1—O4	49.8 (2)
C6—C1—C8—O1	−40.1 (4)	O1—C8—P1—O2	−65.7 (2)
C2—C1—C8—P1	−101.1 (3)	C1—C8—P1—O2	172.18 (19)
C6—C1—C8—P1	80.9 (3)	O1—C8—P1—O3	48.2 (2)
O2—C9—C11—C10	−58.6 (4)	C1—C8—P1—O3	−73.9 (2)
O2—C9—C11—C13	64.1 (4)		

### Hydrogen-bond geometry (Å, °)

**Table:**

*D*—H···*A*	*D*—H	H···*A*	*D*···*A*	*D*—H···*A*
O1—H1···O4^i^	0.80 (5)	1.89 (5)	2.686 (3)	173 (4)

Symmetry codes: (i) −*x*+2, *y*−1/2, −*z*.
